# Causes of death in patients with Berardinelli-Seip congenital generalized lipodystrophy

**DOI:** 10.1371/journal.pone.0199052

**Published:** 2018-06-08

**Authors:** Josivan Gomes Lima, Lucia Helena C. Nobrega, Natalia Nobrega Lima, Marcel Catão Ferreira dos Santos, Pedro Henrique Dantas Silva, Maria de Fatima P. Baracho, Debora Nobrega Lima, Julliane Tamara Araújo de Melo Campos, Leonardo Capistrano Ferreira, Francisco Paulo Freire Neto, Carolina de O. Mendes-Aguiar, Selma Maria B. Jeronimo

**Affiliations:** 1 Departamento de Medicina Clínica, Hospital Universitário Onofre Lopes (HUOL/UFRN), Natal, RN, Brazil; 2 Universidade Federal de Pernambuco (UFPE), Recife, PE, Brazil; 3 Faculdade de Ciências da Saúde do Trairi (UFRN), Santa Cruz, RN, Brazil; 4 Instituto de Medicina Tropical do Rio Grande do Norte (UFRN), Natal, RN, Brazil; Medizinische Fakultat der RWTH Aachen, GERMANY

## Abstract

**Introduction:**

Berardinelli-Seip Congenital Lipodystrophy (BSCL) is a rare autosomal recessive disease that affects the development of adipocytes and leads to an inability to store fat in adipocytes. This study aimed to evaluate the life expectancy and the causes of death of patients with BSCL.

**Method:**

We analyzed death certificates, and medical records of BSCL patients who died between 1997 and 2017. If the death certificate was incomplete or unavailable, we reviewed the medical records, and if they were not available too, we collected information from the patient's relatives to understand how the death happened. We calculated the potential years of life lost as a result of premature death.

**Results:**

Twenty patients (12 female and 8 male) died between 1997 and 2017. The mean age at the time of death was 27.1±12.4 years (women 25.2±12.5 vs. men 29.9±12.6 years, p = 0.41). Life expectancy for the study population was 62.9±4.8 years. The potential number of years of life lost was 35.6±16.6 years. The causes of deaths were divided into three major groups: infections (7 patients, 35%), liver disease (7 patients, 35%), and other causes (acute pancreatitis, one patient; renal failure, three patients; sudden death/myocardial infarction, two patients). Three patients had pulmonary fibrosis.

**Conclusion:**

BSCL led to premature death, cutting the patients’ lifespan by 30 or more years. The majority of these young patients died of liver disease or infection. Other studies are needed to understand better the mechanisms that predispose to infections, as well as to assess whether new therapies can alter the natural history of this disease.

## Introduction

Congenital generalized lipodystrophy or Berardinelli-Seip Congenital Lipodystrophy (BSCL) is an ultra-rare autosomal recessive disease that affects the development of adipocytes and leads to an inability to store excess calories in the form of fat in adipocytes[1]. It was first described by Berardinelli [2] and Seip [3] and is classified based on known mutations in four genes. The most common forms are Type 1 (*AGPAT2* mutations—OMIM 603100) and Type 2 (*BSCL2* mutations—OMIM 606158) [4, 5]. Only a few cases worldwide of Type 3 (*CAV1* mutations—OMIM 601047) and Type 4 (*CAVIN1* mutations—OMIM 603198) have been diagnosed [6, 7].

Non-stored lipids are left in the bloodstream causing hypertriglyceridemia or depositing in ectopic sites, such as in the liver [8]. Patients usually have less than 2% total body fat [9]. This paucity of adipose tissue causes a decrease in leptin and therefore an increase in appetite, which hinders adherence to the diet. Severe insulin resistance causes hypertension and difficulty in controlling diabetes. Fat deposition in the liver can cause cirrhosis. All this taken together explains the severity of this disease that can evolve with early mortality.

It is a rare disease, with a prevalence of approximately 0.96 cases/million and 500 cases described in the literature [1, 10]. Therefore, it is difficult to know the exact causes of deaths in BSCL patients. Even in publications of large series of cases, the number of deaths reported is small [11, 12]. The state of Rio Grande do Norte (RN), Brazil, due to a founder effect (669insA mutation in exon 4 of *BSCL2*) [13], has one of the highest BSCL prevalence rates worldwide [14] More than 50 patients were followed up in the last 20 years in the endocrinology outpatient clinic of Hospital Universitário Onofre Lopes [9]. This study aimed to evaluate the life expectancy and the causes of death of patients with BSCL living in Northeast Brazil.

## Methods

### Data sources

The diagnosis of BSCL was based on the clinical features (absence of subcutaneous adipose tissue, loss of Bichat's fatty ball, prominent veins, acanthosis nigricans, muscular hypertrophy) and laboratory findings (diabetes, hypertriglyceridemia, elevation of transaminases) [9]. Genotyping was conducted in a subgroup of the patients, as previously described [9, 14].

Data of BSCL patients who died between 1997 and 2017, including gender, age, date of death, and the main cause of death, were obtained from death certificates maintained by the Rio Grande do Norte/ Brazil BSCL patient advocacy organization (ASPOSBERN). The certificates had been completed following the “International Model of Medical Certificate of the Cause of Death” used in all countries and recommended by the World Health Organization [15]. Necropsy was performed in one patient.

If the death certificate was incomplete, without a clear cause of death, or if it was not available, we reviewed the medical records, and if they were not available too, we collected information from the patient's relatives, after obtaining the informed consent of them. Patients who died of respiratory failure after pneumonia were counted as infection/respiratory failure. If the main cause of death was gastrointestinal bleeding or liver failure due to cirrhosis, it was recorded as liver disease. Myocardial infarction and sudden death were counted in the same group.

We individually calculated the potential years of life lost as a result of premature death. For this calculation, life expectancy was considered for Brazilians in the year each patient was born. The prediction was made subtracting the patient's age at death from the reference age. This reference population was obtained from the World Bank database [16].

### Statistical analyses

Descriptive analysis was used to characterize the population. Mean and standard deviation were used for parametric variables. We compared means of the groups using t-Test or ANOVA. A value of p <0.05 was considered statistically significant. SPSS version 22.0 was used for statistical analysis.

## Results

### Patients

Of the 20 patients (12 female and 8 male) who died with BSCL between 1997 and 2017, 16 had a death certificate available. All patients (n = 12) for which we performed genotyping had the 325dupA variant in the BSCL2 gene (rs786205071) and all but one patient at the time of death had diabetes. Table 1 describes all cases collected. None of the patients used or had used leptin replacement therapy (metreleptin).

**Table 1:** Causes of death and individual clinical features of patients with Berardinelli-Seip Congenital Lipodystrophy.

Patient#	Sex	BSCL type	Year of death	Age at death (years)	Reported main cause of death	Associated comorbidities
1	Male	NA†	1997	11	Liver disease	Diabetes
2	Male	NA	1997	21	Liver disease	Diabetes, kidney failure
3	Male	NA	1998	29	Respiratory insufficiency	Diabetes, pulmonary fibrosis
4	Female	NA	1999	27,1	Liver disease	Diabetes
5	Male	NA	2000	29	Gastrointestinal bleeding	Diabetes, cirrhosis
6	Female	NA	2002	9.3	Sepsis, pneumonia	Diabetes
7	Female	2	2005	39.7	Sepsis, pneumonia	Diabetes, cirrhosis, kidney failure
8	Female	NA	2009	27.6	Septic arthritis	Diabetes, cirrhosis, kidney failure (hemodialysis)
9	Female	2	2011	43.9	Kidney failure	Diabetes, cirrhosis, kidney failure, pulmonary fibrosis
10	Female	2	2011	21.5	Liver disease	Diabetes
11	Male	2	2013	41.7	Respiratory insufficiency	Diabetes, pulmonary fibrosis
12	Male	2	2013	20.4	Myocardial infarction*	Diabetes, acute pulmonary edema, arterial hypertension
13	Male	2	2013	52.6	Sudden death	Diabetes
14	Male	2	2014	25.8	Gastrointestinal bleeding	Diabetes, perforated gastric ulcer, kidney failure (hemodialysis)
15	Female	2	2014	18	Acute pancreatitis	Diabetes
16	Female	2	2014	31	Gastrointestinal bleeding	Diabetes
17	Female	NA	2015	2.1	Pneumonia	-
18	Female	2	2015	29.5	Sepsis, pneumonia	Diabetes, kidney failure
19	Male	2	2016	29.5	Kidney failure	Diabetes, amaurosis, hemodialysis
20	Female	2	2016	40.2	Kidney failure	Diabetes, amputation of leg, anemia

* Confirmed by necropsy.

Patients #1 and #2, and #3 and #11 are brothers. Patients #9 and #20 are sisters. Patient #6 is the sister of #14.

†NA = not available.

### Age and year of death

**Fig 1:**
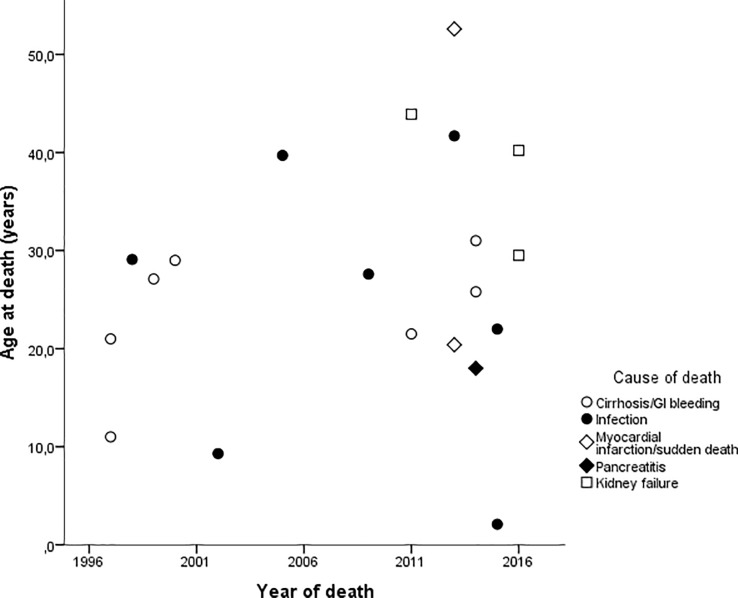
Age at death (years-old) of Berardinelli-Seip Congenital Lipodystrophy patients according to the year of death.

The mean age at the time of death was 27.1±12.4 years. Women tended to die earlier than men (25.2±12.5 vs. 29.9±12.6 years of age, p = 0.41). Although not statistically significant, the data suggested a slightly greater longevity in the most recent years (the 1990s, 22.1±8.1 years; the 2000s, 26.4±12.6 years; the 2010s, 29.1±13.8 years, p>0.05). Most deaths evaluated occurred after 2010 (n = 12), four deaths occurred in the 1990s and another four in the 2000s (Fig 1).

Life expectancy for the study population was 62.9±4.8 years. The potential number of years of life lost was 35.6±16.6 years. Patients who died of infection or liver disease lost more years of life (39.0±20.3 years, and 39.2±8.6 years, respectively).

### Causes of death

**Fig 2:**
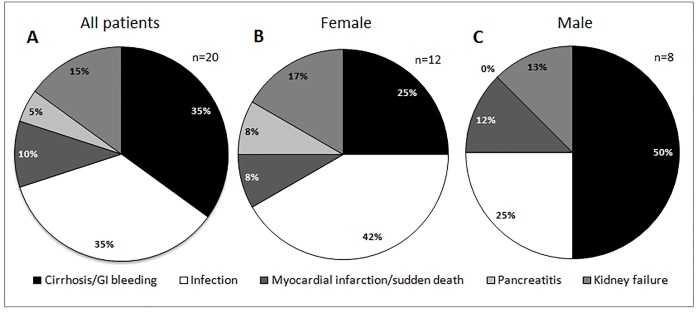
Main causes of death in Berardinelli-Seip Congenital Lipodystrophy patients. Results divided by gender: female (B), and male (C).

We divided the causes of deaths into three major groups: infections, liver disease, and other causes (acute pancreatitis, renal failure, sudden death/myocardial infarction) (Fig 2). Except for the youngest patient (who died of infectious cause), all patients already had diabetes.

Death due to infection occurred in seven patients (35%). Pneumonia was the most frequent cause in this group, occurring in four patients (#6, 7, 17, and 18). Two patients (#3 and 11) had respiratory failure and imaging tests compatible with pulmonary fibrosis. One of these patients had a positive family history of tuberculosis and, despite the negative results of the direct examination of the sputum, he was treated for tuberculosis, without apparent clinical benefit. The last patient in the infection group (#8) died due to cellulitis in the right thigh that evolved with incomplete muscle rupture and septic arthritis.

Another seven patients (35%) died due to liver disease or its consequences. Three patients (#5, 14, and 16) died after upper gastrointestinal bleeding due to cirrhosis. Four patients (#1, 2, 4, and 10) had advanced liver disease and died from liver failure.

Two patients (#12 and 13) had sudden death; one of them was submitted to necropsy, which confirmed acute myocardial infarction. Only one patient (#15) died due to acute pancreatitis.

Patient #19 had chronic renal failure and was decompensated, requiring hemodialysis more frequently. He died when he was going to a hemodialysis session. Although it was a sudden death, the cause of his death was attributed to kidney failure. Another patient (#20) had severe anemia and also underwent hemodialysis; she died due to renal failure. Patient #9, like patients #3 and 11, also had pulmonary fibrosis and had hospitalizations with respiratory decompensation (with antibiotic treatment), but she died due to a hydroelectrolytic disorder caused by renal failure (according to the death certificate).

**Fig 3:**
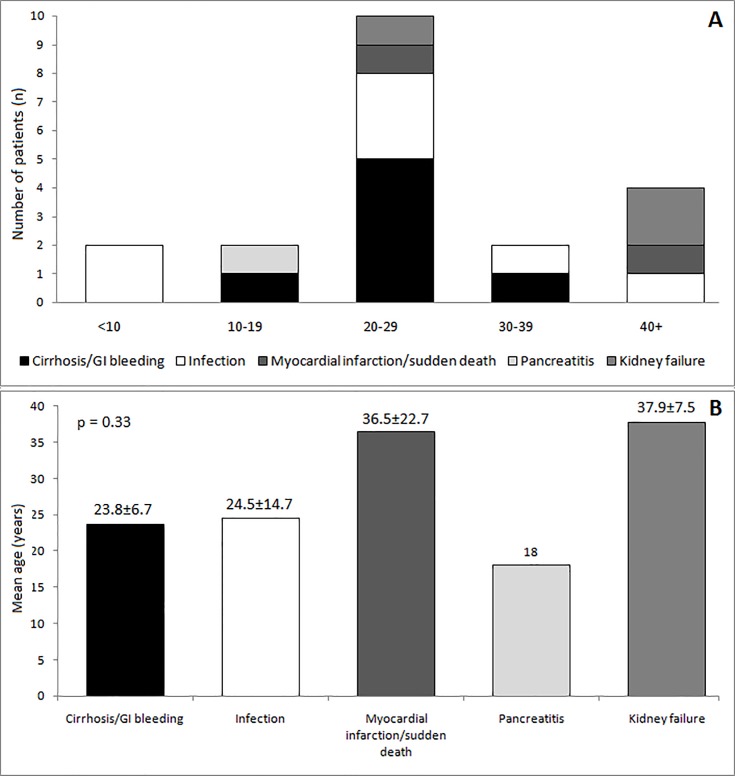
(A) Causes of death (n) stratified according to the age group of patients. (B) Patients’ mean age according to the cause of death.

The mean age of death in each group was: liver disease, 23.8±6.7; infection, 24.5±14.7; sudden death, 36.5±22.7; and renal failure, 37.9±7.5 years old (p = 0.33). Only one patient, an 18-year-old female, died of acute pancreatitis; her triglyceridemia at the time of death was not available, but since hypertriglyceridemia is a frequent finding of the syndrome, we believe that this was the cause of pancreatitis. The only two patients less than ten years old died of infection. All patients who died as a result of renal failure were older than 29 years (Fig 3).

## Discussion

As far as we know, this is the most extensive study to report the causes of death in adults and children with BSCL. Because it is a rare disease, it is not usual to have so many patients followed in one single clinical setting, and it is even rarer to report the deaths of so many patients. Even when reporting patients from different countries together, the number of deaths is small, making it difficult to know the exact causes of death [11, 12]. Our patients used only conventional treatments for diabetes, hypertension, and dyslipidemia. None of them was on treatment with leptin (metreleptin), so we believe this is the natural history of the disease.

Most deaths occurred after 2010, which probably indicates improvement in case detection. One of the patients (#19) who died most recently had been diagnosed with diabetes since adolescence but was only diagnosed with BSCL two years before death.

The average age of death is low, indicating the severity of BSCL, causing a loss of more than 30 years of life. Extreme insulin resistance and diabetes are inescapable in these patients and likely contribute to this severity. Although not statistically significant (small sample?), the longevity has improved over the years, possibly due to improvements in therapeutic options and access to specialized health care.

Deaths due to chronic complications of diabetes (renal failure, myocardial infarction) or liver disease and its consequences (cirrhosis, upper gastrointestinal bleeding) were expected in this group of patients. However, one-third of the patients died from infectious diseases. Recurrent infections requiring hospitalizations is not uncommon in the patients in our clinic. We often attributed this to poor glycemic control predisposing to infections. However, the youngest patient in our series had no diabetes and died of infectious disease, challenging our hypothesis.

The brother of two other patients followed in our service (not included here due to lack of detailed clinical data) died at four years of age in the 1980s due to diphtheria infection. His relatives reported that he had the lipodystrophy phenotype, but he did not have diabetes. Since children usually have not yet developed diabetes or liver disease, infection appears to be the most frequent cause of death in childhood [11].

Van Maldergem *et al*. [12] reported genotype and phenotype of patients with BSCL. From their series, no BSCL type 1 and 7 BSCL type 2 patients died; 2 (28.5%) of them were due to infection (pneumonia and sepsis). As in our series, the mean age of death was 26.2±4.7 years, and the youngest patient (4 months) died from infection (sepsis). Gupta *et al*. [11], evaluating BSCL patients diagnosed during childhood, reported 15 patients with a known cause of death. Of these, 6 (40%) died due to respiratory infection and 1 due to peritonitis. Rheuban *et al*. [17] reported a case of a patient who died of peritonitis and septicemia at 23 years of age. Two of the four deaths reported by Bjornstad *et al*. [18] also died due to lung infection; the mean age of death was 32 years. Hsu R-H *et al*. [19] reported a series of 16 pediatric patients with BSCL type 2 in Taiwan; 3 of these patients died with an average age of 10.9 years. One died from arrhythmia, another from bilateral pneumonia, and the third from an unknown cause. All this together with the data reported by us indicates that patients with BSCL die early and infection (mainly pulmonary) is an important cause of death.

Regarding potential mechanism of infection in these patients, there are leptin receptors on macrophages, and the leptin stimulates the phagocytic activity of these cells [20]. Low serum leptin levels in BSCL patients could predispose to infections. Disruption in membrane lipids or in lipid rafts facilitating the entry of intracellular parasites or causing defective antigen presentation could also be potential mechanisms [21]. HDL cholesterol below 31 mg/dL is associated with increased predisposition to infections [22] Most BSCL patients have low HDL [9] and this could also contribute to the increased risk for infections.

Three patients had pulmonary fibrosis. This complication has never been reported in the literature, and we do not know whether this could be due to lipodystrophy or an associated disease.

Usually, triglyceridemia should be higher than 1000 mg/dL to develop acute pancreatitis [23]. Although hypertriglyceridemia is frequent in BSCL, such high values were not usual in our cohort as well as others [9, 24]. Acute pancreatitis did not happen as often in our patients and was the cause of death in only one patient in our cohort.

Our study has some limitations. A necropsy was performed on only one patient, and most of the data were extracted from death certificates. However, the causes reported on death certificates usually have a good concordance with the actual cause of death, even without a necropsy [25]. The number of patients is small, but we have to remember this is a rare disease [1, 10]. All patients who had the mutation identified were BSCL Type 2, and maybe our data should not be extrapolated to patients with other types of BSCL. However, when we evaluate the deaths already reported in the literature [11, 12, 17, 18], infection is still responsible for more than a third of the cases (13 out of 32, 40.6%). BSCL type 1 is less severe and may not have the same severity or causes of death. At our hospital [9], we only follow four BSCL Type 1 patients, and they appear to have a better prognosis (personal observation). It is also important to note that none of the deaths reported by Van Maldergem *et al*. [12] was Type 1. We do not know whether the loss of years of life can be applied to patients from other countries. However, the mean age at death of patients from different countries is similar to ours [12]. Since life expectancy in developed countries is higher than in Brazil, the number of years of life lost could be even higher in these countries.

In conclusion, this is the most extensive series reporting the causes of death in BSCL patients. This syndrome causes premature death, cutting patients´ lifespan by 30 or more years. The majority of these young patients die of liver disease and infection. Other studies are needed to understand better the exact mechanisms that cause the deaths, as well as to assess whether new therapies, such as metreleptin, can alter the natural history of this disease.

## Supporting information

S1 Table Clinical data of patients and cases reported in the literature. (DOCX)

## Abbreviations

BSCL: Berardinelli-Seip Congenital Lipodystrophy
