# Supplementary material for: Causes of death in patients with Berardinelli-Seip congenital generalized lipodystrophy
Source: PLoS One. 2018 Jun 8;13(6):e0199052. doi: 10.1371/journal.pone.0199052 (PMC5993255; doi:10.1371/journal.pone.0199052)
Supplement: S1 Table — (DOCX) [file pone.0199052.s001.docx]

Clinical data of the 20 patients studied and reported.

| **Patient#** | **Sex** | **BSCL type** | **Year of death** | **Age at death (years)** | **Main cause of death** | **Associated comorbidities** |
| --- | --- | --- | --- | --- | --- | --- |
| 1 | Male | NA† | 1997 | 11 | Liver disease | Diabetes |
| 2 | Male | NA | 1997 | 21 | Liver disease | Diabetes, kidney failure |
| 3 | Male | NA | 1998 | 29 | Respiratory insufficiency | Diabetes, pulmonary fibrosis |
| 4 | Female | NA | 1999 | 27,1 | Liver disease | Diabetes |
| 5 | Male | NA | 2000 | 29 | Gastrointestinal bleeding | Diabetes, cirrhosis |
| 6 | Female | NA | 2002 | 9.3 | Sepsis, pneumonia | Diabetes |
| 7 | Female | 2 | 2005 | 39.7 | Sepsis, pneumonia | Diabetes, cirrhosis, kidney failure |
| 8 | Female | NA | 2009 | 27.6 | Septic arthritis | Diabetes, cirrhosis, kidney failure (hemodialysis) |
| 9 | Female | 2 | 2011 | 43.9 | Kidney failure | Diabetes, cirrhosis, kidney failure, pulmonary fibrosis |
| 10 | Female | 2 | 2011 | 21.5 | Liver disease | Diabetes |
| 11 | Male | 2 | 2013 | 41.7 | Respiratory insufficiency | Diabetes, pulmonary fibrosis |
| 12 | Male | 2 | 2013 | 20.4 | Myocardial infarction* | Diabetes, acute pulmonary edema, arterial hypertension |
| 13 | Male | 2 | 2013 | 52.6 | Sudden death | Diabetes |
| 14 | Male | 2 | 2014 | 25.8 | Gastrointestinal bleeding | Diabetes, perforated gastric ulcer, kidney failure (hemodialysis) |
| 15 | Female | 2 | 2014 | 18 | Acute pancreatitis | Diabetes |
| 16 | Female | 2 | 2014 | 31 | Gastrointestinal bleeding | Diabetes |
| 17 | Female | NA | 2015 | 2.1 | Pneumonia | - |
| 18 | Female | 2 | 2015 | 29.5 | Sepsis, pneumonia | Diabetes, kidney failure |
| 19 | Male | 2 | 2016 | 29.5 | Kidney failure | Diabetes, amaurosis, hemodialysis |
| 20 | Female | 2 | 2016 | 40.2 | Kidney failure | Diabetes, amputation of leg, anemia |

This is the Table 1 legend. * Confirmed by necropsy. Patients #1 and #2, and #3 and #11 are brothers. Patients #9 and #20 are sisters. Patient #6 is the sister of #14. †NA = not available.

Cases reported in the literature.

| **Author (year)** | **n** | **Patient#** | **Gender** | **Age of death** | **Heart failure** | **Infection** | **Liver disease** | **Kidney failure** | **AMI** | **Arrythmia** | **Acute pancreatitis** | **Multi organ failure** | **Epilepsy** |
| --- | --- | --- | --- | --- | --- | --- | --- | --- | --- | --- | --- | --- | --- |
| Van Maldergem (2002) | 8 | 4 | Male | 14 | 1 |  |  |  |  |  |  |  |  |
|  |  | 8 | Female | 32 |  | 1 |  |  |  |  |  |  |  |
|  |  | 10 | Male | 35 |  |  |  | 1 |  |  |  |  |  |
|  |  | 12 | Female | 24 | 1 |  |  |  |  |  |  |  |  |
|  |  | 21 | Female | 0,3 |  | 1 |  |  |  |  |  |  |  |
|  |  | 26 | Female | 31 |  |  | 1 |  |  |  |  |  |  |
|  |  | 31 | Male | 31 | 1 |  |  |  |  |  |  |  |  |
|  |  | 1 | Female | 7,5 |  |  | 1 |  |  |  |  |  |  |
| Gupta (2017) | 15 |  |  | 12,5 | 3 | 7 | 1 | 1 |  |  |  | 1 | 2 |
| Bjmnstad (1996) | 4 | 1 | Female | 32 |  | 1 |  |  |  |  |  |  |  |
|  |  | 3 | Male |  |  |  |  |  | 1 |  |  |  |  |
|  |  | 5 | Female |  |  |  |  |  | 1* |  |  |  |  |
|  |  | 8 | Male | 24 |  | 1 |  |  |  |  |  |  |  |
| Rheuban (1986) | 1 | 1 | Female | 23 |  | 1 |  |  |  |  |  |  |  |
| Rai-Hseng Hsu (2018) | 3 | 1 | NA | 11,6 |  |  |  |  |  | 1 |  |  |  |
|  |  | 2 | NA | 9,1** |  |  |  |  |  |  |  |  |  |
|  |  | 3 | NA | 12 |  | 1 |  |  |  |  |  |  |  |
| Total | 31 |  |  | 19,9 | 6 | 13 | 3 | 2 | 2 | 1 | 0 | 1 | 2 |
| % |  |  |  |  | 19,4% | 41,9% | 9,7% | 6,5% | 6,5% | 3,2% | 0,0% | 3,2% | 6,5% |

* Found dead in her home. **Unknow cause. AMI = Acute myocardial infarction
